# Analgesic Effect of *Harpagophytum procumbens* on Postoperative and Neuropathic Pain in Rats

**DOI:** 10.3390/molecules19011060

**Published:** 2014-01-16

**Authors:** Dong Wook Lim, Jae Goo Kim, Daeseok Han, Yun Tai Kim

**Affiliations:** Functionality Evaluation Research Group, Korea Food Research Institute, Seongnam 463-746, Korea

**Keywords:** *Harpagophytum procumbens*, pain, hyperalgesia, allodynia, ultrasonic vocalization

## Abstract

*Harpagophytum procumbens*, also known as Devil’s Claw, has historically been used to treat a wide range of conditions, including pain and arthritis. The study was designed to investigate whether *H. procumbens* extracts exhibit analgesic effects in plantar incision and spared nerve injury (SNI) rats. The whole procedure was performed on male SD rats. To evaluate pain-related behavior, we performed the mechanical withdrawal threshold (MWT) test measured by von Frey filaments. Pain-related behavior was also determined through analysis of ultrasonic vocalization (USVs). The results of experiments showed MWT values of the group that was treated with 300 mg/kg *H. procumbens* extract increased significantly; on the contrary, the number of 22–27 kHz USVs of the treated group was reduced at 6 h and 24 h after plantar incision operation. After 21 days of continuous treatment with *H. procumbens* extracts at 300 mg/kg, the treated group showed significantly alleviated SNI-induced hypersensitivity responses by MWT, compared with the control group. These results suggest that *H. procumbens* extracts have potential analgesic effects in the case of acute postoperative pain and chronic neuropathic pain in rats.

## 1. Introduction

Pain is a common symptom but very distressing feature of many diseases. In general, analgesics relieve pain by acting on the central nervous system or peripheral pain mechanisms, without significantly altering consciousness [1]. Patients suffering from this kind of pain include those with so-called nociceptive and neuropathic pain, allodynia, and hyperalgesia. [2,3]. However, pain management remains a major clinical challenge, because there is not an appropriate understanding of the mechanisms causing and maintaining pain and effective treatments [4].

Therapeutic drugs for treating pain have limited effectiveness and safety [5]. Repetitive use of non-steroidal anti-inflammatory drugs (NSAIDs) may cause adverse effects such as gastrointestinal lesions or renal and liver failure [6]. Furthermore, current analgesic drugs, even including the opioids, cannot ease the pain easier of painful conditions like neuropathic pain [7]. Therefore, it is necessary to search for new, effective and safe analgesics among natural products derived from secondary metabolites. Natural products are considered an incomparable source of molecular diversity that has led to the discovery of drugs in modern medicine, especially for pain treatment [8,9,10].

*Harpagophytum procumbens*, also known as Devil’s Claw, is a herbaceous plant species that has the high level of medicinal use value in the Kalahari Desert region of southern Africa [11]. It has historically been used to treat several symptoms such as fever, malaria, indigestion and pain [12]. In addition, it was demonstrated that *H. procumbens* extracts have beneficial effects in the case of rheumatic diseases according to animal and clinical studies [13]. There have been reports verifying the anti-inflammatory effects of *H. procumbens* extracts on acute or sub-chronic inflammation in a rat model [14,15]. However, no studies have been made of the effect of *H. procumbens* extracts on surgical incision postoperative pain or neuropathic pain in *in vivo* models. Therefore, the present study was designed to investigate whether *H. procumbens* extracts exhibit anti-nociceptive effects in the postoperative pain through plantar incision model [16] and on the spared nerve injury (SNI) rat model of neuropathic pain [17]. To evaluate pain-related behavior, we performed the mechanical withdrawal threshold (MWT) test measured by von Frey filaments, and pain-induced ultrasonic vocalizations (USVs) have been examined by ultrasonic microphones [18]. Further, the results have been compared with those of naproxen, a NSAID [19].

## 2. Results and Discussion

### 2.1. Effects of H. Procumbens Extracts on Mechanical Hyperalgesia Induced by Plantar Incision

**Figure 1:**
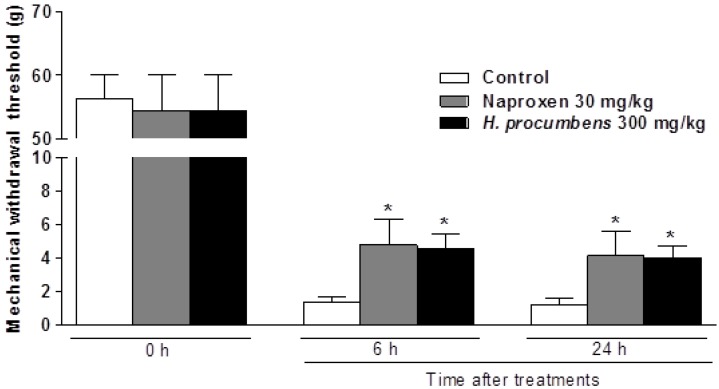
Effect of *H. procumbens* extracts on mechanical hyperalgesia induced by plantar incision in rats. Baseline assessment of animals, before surgery (day 0), showed no significant variation between groups. 6 h or 24 h after surgery, rats treated with *H. procumbens* extracts significantly attenuated hyperalgesia in response to von Frey stimulation of injured hind paw. Data are mean ± SEM (n = 6 per group). * *p* < 0.05, significant difference from the control group.

The analgesic activity of *H. procumbens* extracts was determined using the postoperative pain model in rats. Postoperative pain in humans can be mimicked by plantar incision in rats [16]. Incision of the plantar surface of the hind paw produced a significant reduction in the mechanical withdrawal threshold (MWT), as measured using the von Frey assay, and non-steroidal anti-inflammatory drugs (NSAIDs). including naproxen, effectively reverse incision-induced decreases in the MWT against mechanical hyperalgesia [20]. The plantar incision produced a marked mechanical hyperalgesia in the incised paw (paw withdrawal threshold diminished from 56.22 ± 11.35 g at baseline to 1.17 ± 1.29 g 24 h after plantar incision; *p* < 0.001). Administration of *H. procumbens* extracts (300 mg/kg, p.o.) significantly attenuated hyperalgesia in response to von Frey stimulation of the injured hind paw as evidenced by an increased mechanical withdrawal threshold (MWT) values as compared to control rats (4.00 ± 1.78 g *vs.* 1.17 ± 1.29 g, *p* < 0.05) 24 h after incision surgery. The group treated with 300 mg/kg of *H.procumbens* extracts showed similar results as the group treated with 30 mg/kg of naproxen (Figure 1).

### 2.2. Effects of H. Procumbens Extracts on Ultrasonic Vocalizations (USVs) Induced by Plantar Incision

**Figure 2:**
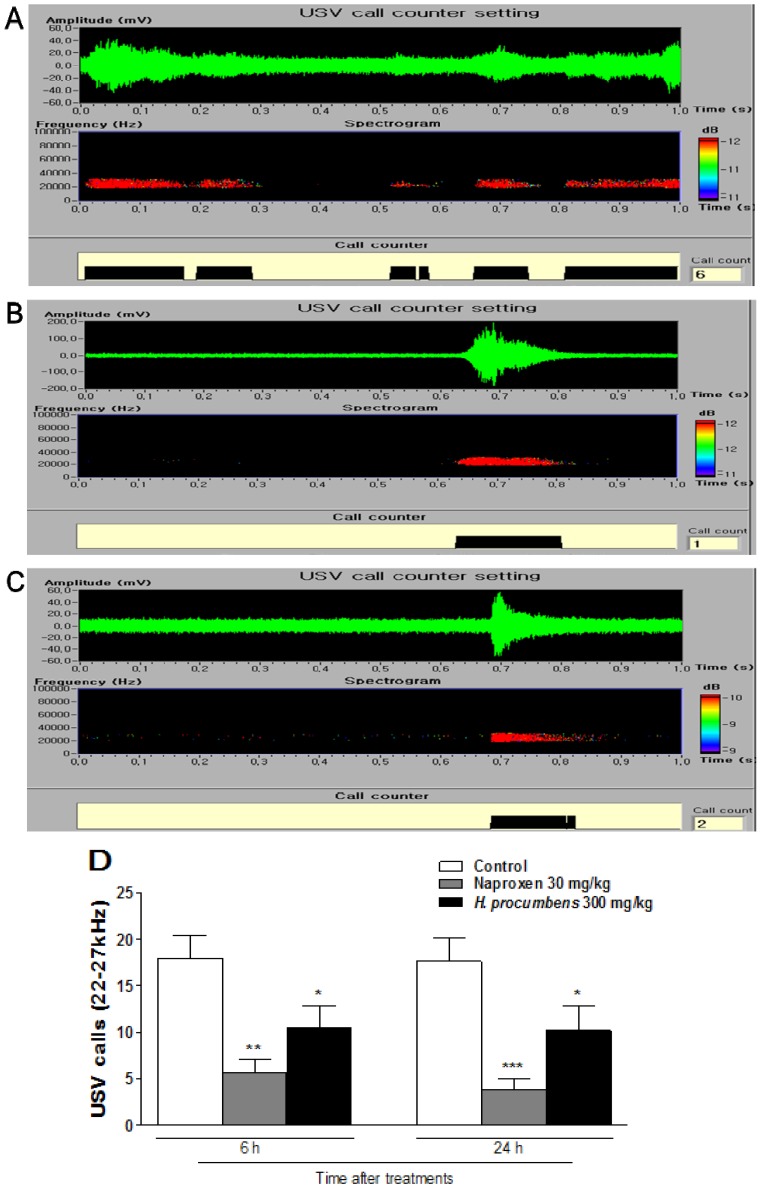
Effect of *H. procumbens* extracts on USVs induced by plantar incision in rats. The sonograms of USVs in (**A**) control, (**B**) naproxen- and (**C**) *H. procumbens* extract- treated rats. (**D**) A significant difference in 22–27 kHz USVs was observed between the *H. procumbens* extracts (300 mg/kg) treated group and the control group. Data are mean ± SEM (n = 6 per group). * *p* < 0.05, significant difference from the control group.

The anti-nociceptive activity of *H. procumbens* extracts was also determined by the pain-induced ultrasonic vocalizations (USVs) using ultrasonic microphones. Adult rats produce two distinct types of USVs that appear to reflect the caller’s emotional state: either positive (a high-pitched and short ~50 kHz USVs) or negative state (a low-pitched and longer ~27 kHz USV) [21]. In particular, 22–27 kHz USVs have been suggested as being a measure of affective shifts in rats [22] and have been used in a variety of unconditioned models such as pain, anxiety and stress-related models [23,24]. Pain-induced USVs have been examined by ultrasonic microphones, because vocalization is not only an objective but also a quantifiable value in the rats. After 6 h or 24 h after plantar incision, the control group emitted 22–27 kHz USVs calls, in pain-related behaviors [25]. The group treated with 30 mg/kg of naproxen showed significantly reduced 22–27 kHz USV calls compared with the control group, showing its anti-nociceptive effects in rats. *H. procumbens* extracts also reduced 22–27 kHz USVs; a significant reduction was observed after the administration of *H. procumbens* extracts at 300 mg/kg (73.5%, *p* < 0.01 *vs.* control) (Figure 2). According to our findings, compared to the experimental group treated with naproxen, the *H. procumbens* extrcts might have anti-nociceptive effects on plantar incision postoperative pain in rats.

### 2.3. Effects of H. Procumbens Extracts on Mechanical Allodynia Induced by Spared Nerve Injury (SNI)

**Figure 3:**
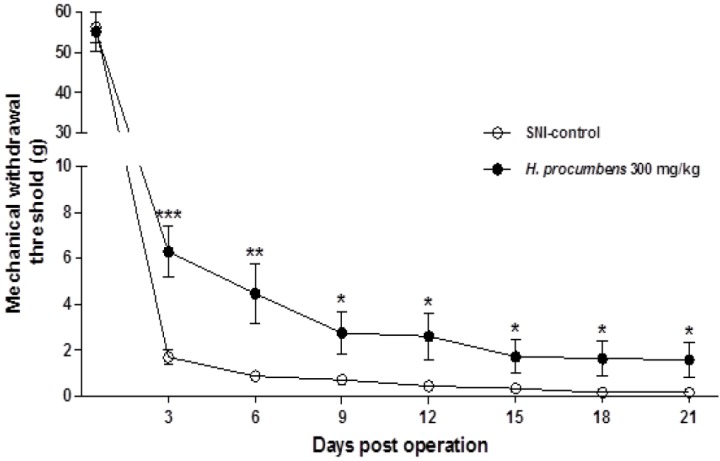
Anti-allodynic effects of *H. procumbens* extracts treatment in SNI rat model of neuropathic pain. Administration of *H. procumbens* extracts (300 mg/kg, p.o) significantly attenuated allodynia in response to von Frey stimulation of hind paw from 3 to 21 day after treatment. Data are mean ± SEM (n = 6 per group). *** *p* < 0.001, ** *p* < 0.01, and * *p* < 0.05 significant difference from control group.

In this study we evaluated the potential efficacy of *H. procumbens* extracts in a rat model of the spared nerve injury (SNI) with regard to neuropathic pain. SNI mimics the symptoms of chronic nerve compression in humans [26]. At baseline (day 0), there are no significant changes between the *H. procumbens* extract (300 mg/kg)-treated group and SNI-control group (Figure 3). Animals began to show hypersensitivity responses to von Frey stimulation 3 days of after operation during the experiments. The frequency of withdrawals in the SNI-control group increased significantly reached from 56.22 ± 3.77 g day 0 to 0.14 ± 0.05 g on day 21 after SNI-operation. Administration of *H. procumbens* extracts (300 mg/kg) significantly attenuated allodynia in response to von Frey stimulation of the hind paw, as evidenced by increased MWT values as compared to SNI-control rats from 3 to 21 day after treatment, with an inhibition of 83.6% (*p* < 0.001).

Wells *et al.* demonstrated that TNF (tumor necrosis factor) is increased in plasma after nerve crushing [27], and endoneurial injection of TNF-α can produce neuropathic pain behavior [28]. Also, Cyclooxygenase-2 (COX-2) is one of the important rate-limiting enzymes in the synthesis of prostaglandin (PG), a pain inducing substance, and is also the target of many non-steroidal analgesics. A study has showed significantly increased COX-2 positive cells on the first day and from day 7 to 14 following nerve injury, which is associated the formation of neuropathic pain [29]. It has been reported that both an ethanolic extract and an aqueous extract of *H. procumbens* prevented TNF-α synthesis; the latter, however, having a greater inhibitory effect on COX-2 pathway products [30,31]. Taken together, it might be hypothesized that *H. procumbens* extracts attenuated the behavioral symptoms of neuropathic pain in rats.

## 3. Experimental

### 3.1. Preparation of H. Procumbens Extracts

*H. procumbens* (300 g) was extracted with 70% ethanol (3 L) for 4 h at 80 °C in a reflux apparatus. The process was repeated twice, and the extracts were filtered through membrane filters (0.45 µm; Millipore, Billerica, MA, USA). The samples were lyophilized to yield a dark yellow powder. The yield of *H. procumbens* extracts was 41.6%.

### 3.2. Animals and Treatments

Male Sprague-Dawley (SD) rats (160–200 g) were purchased from Samtako (Gyeonggi-do, Korea). Animals were housed at two rats per cage in an air-conditioned room at 23 ± 1 °C, 55%–60% relative humidity, and a 12 h light/dark cycle (07:00 lights on, 19:00 lights off), and were given a regular laboratory rodent diet. After acclimatization for 1 week, 8-week-old male SD rats were anesthetized with 2% of isoflurane and pain-related surgeries were performed. After plantar incision operation, rats were divided into three following treatment groups: (1) control + vehicle, (2) control + naproxen (30 mg/kg, i.p.), and (3) control + *H. procumbens* extracts (300 mg/kg, p.o.). *H. procumbens* extracts and naproxen were dissolved in distilled water for oral administration at the desired doses in a volume of 5 mL/kg. The dosage of *H. procumbens* extracts was selected in consideration of usual human dosages (1.5–9 g/60 kg-weighted human, extracts dosages) commonly used in herbal medicine practice [12,32]. *H. procumbens* extracts at a dosage of 300 mg/kg in rats corresponds to 1.8 g *H. procumbens* extracts/60 kg-weight human subject, corresponding to *H. procumbens* extracted from approximately 4.3 g of the *H. procumbens* raw material. The sample treated groups were oral administrated *H. procumbens* extracts or naproxen after the plantar incision operations. After spared nerve injury (SNI) operation, rats were divided into two following treatment groups: (1) SNI-control + vehicle, and (2) SNI-control + *H. procumbens* extracts (300 mg/kg). *H. procumbens* extracts was given by p.o. route, immediately following surgery, once a day, which continued for 21 consecutive days. All animal experiments were carried out according to the guidelines of the Korea Food Research Institutional Animal Care and Use Committee.

### 3.3. Plantar Incision of Postoperative Pain Rat Model

Surgery was performed as previously described [16], with minor modifications. Briefly, rats were anaesthetized with 2% isofluorane and, a 1 cm longitudinal incision was made with scalpel, through skin and fascia of the plantar aspect of the paw, starting 0.5 cm from the proximal edge of the heel and extending toward the toes. The plantar muscle was raised and incised longitudinally. Following haemostasis with gentle pressure, the skin was opposed with two single interrupted sutures using polyamide monofilaments. The animals were allowed to recover in their home cages.

### 3.4. Spared Nerve Injury (SNI) of Neuropathic Pain Rat Model

The surgical procedure was performed as described previously with some modifications [33]. The SNI procedure comprised an axotomy and ligation of the tibial and common peroneal nerves leaving the sural nerve intact. The common peroneal and the tibial nerves were tightly ligated with 5.0 silk and sectioned distal to the ligation, removing 2 ± 4 mm of the distal nerve stump. Great care was taken to avoid any contact with or stretching of the intact sural nerve. The skin was opposed with two single interrupted sutures using polyamide monofilaments.

### 3.5. Mechanical Withdrawal Threshold (MWT) Analysis

Animals were placed on an elevated wire grid and the plantar surface of the paw stimulated with a series of ascending force von Frey monofilaments (Stoelting, Chicago, IL, USA). The threshold was taken as the lowest force that evoked a brisk withdrawal response to one of three repetitive stimuli. To determine the time course of hyperalgesia and allodynia, a baseline measurement was made prior to surgery, and then again at 6 and 24 h post-surgery for hyperalgesia, 3, 6, 9, 12, 15, 18, and 21 days post-surgery for allodynia.

### 3.6. Ultrasonic Vocalization (USVs) Analysis

After induction of the plantar incision of postoperative pain, the category of 22–27 kHz USVs emitted by adult rats was monitored and scored for 10 min using Sonotrack ultrasonic microphones (Metris B.V., KA Hoofddorp, The Netherlands) placed at a distance of 25–30 cm from the heads of the animals. The rats emitted ‘calls’ that were counted using the Sonotrack 2.2.1 software.

### 3.7. Statistical Analysis

Data analyses were performed using one-way analysis of variance (ANOVA), followed by Tukey’s post hoc test, using Prism 5 (GraphPad Software, Inc., San Diego, CA, USA) for multigroup comparisons. All data are presented as the mean ± standard error (SEM). Significance was set at *p* < 0.05.

## 4. Conclusions

In conclusion, the analgesic effect of *H. procumbens* extracts led to a reduction in the number of ultrasonic distress vocalizations caused by plantar incision postoperative pain in rats, and to a decrease in allodynia in response to von Frey stimulation of the hind paw evidenced by a decreased mechanical withdrawal threshold (MWT) in the spared nerve injury (SNI) neuropathic pain rat model. These results suggest that *H. procumbens* extracts could be useful on the treatment of postoperative and neuropathic pain, but more pharmacological and toxicological investigations are needed for finding the exact mechanism of action and for safety evaluation.
